# COVID-19 Information Overload, Negative Emotions and Posttraumatic Stress Disorder: A Cross-Sectional Study

**DOI:** 10.3389/fpsyt.2022.894174

**Published:** 2022-05-13

**Authors:** Jingjun Wang, Xia Huang, Ya Wang, Mengmeng WANG, Jiajun XU, Xiaolin LI

**Affiliations:** ^1^West China School of Nursing and West China Hospital, Sichuan University, Chengdu, China; ^2^Department of Nursing, West China Hospital and West China School of Nursing, Sichuan University, Chengdu, China; ^3^School of Nursing, Peking University, Beijing, China; ^4^Mental Health Center, West China Hospital, Sichuan University, Chengdu, China

**Keywords:** information dissemination, posttraumatic stress disorder, depression, anxiety, mental health, nursing, COVID-19

## Abstract

**Background:**

Under the context of the COVID-19 pandemic, a large amount of COVID-19-related information can cause an individual's perceived information overload, further halting the individual's psychological health. As a minor psychological discomfort could develop severe mental disorders such as posttraumatic stress disorder, it is necessary to understand the chain linkage of COVID-19 information overload turn to posttraumatic stress disorder to ensure timely intervention can be offered at each point of mental state transformation. Hence, we examined the negative outcomes of COVID-19 information overload and investigated the direct and indirect effects of COVID-19 on posttraumatic stress disorder.

**Methods:**

A convenient sample of Chinese adults (*n* = 1150) was investigated by an online survey from July 2020 to March 2021. The extent of COVID-19 information overload was measured by the information overload severity scale on the text of the COVID-19 pandemic. Psychological distress symptoms were measured using a 7-item anxiety scale (GAD-7), the 9-item Patient Health Questionnaire depression module (PHQ-9), and the psychometric properties of the PTSD Checklist (PCL-C). Structural equation modeling and bootstrap methods were utilized to analyze the relationships between variables.

**Results:**

COVID-19 information overload is positively related to an individual's anxiety, depression, and posttraumatic stress disorder. Furthermore, COVID-19 information overload can indirectly affect an individual's PTSD symptoms by increasing the feeling of depression. R^2^ values of anxiety, depression, and PTSD were 0.471, 0.324, and 0.795, respectively.

**Conclusion:**

COVID-19 information overload, anxiety, depression, and PTSD are negative psychological states, and each variable is closely linked with the others, suggesting the need for potential psychological interventions at specific times. Practical public training, such as crisis coping and information filtering, is essential. Regulation of technology companies is also essential.

## Introduction

In December 2019, the first evidence of the novel coronavirus disease (COVID-19) was found in China. Within a short period of time, the disease spread rapidly throughout the world, and by January 2022, there had been more than 38 million cases of COVID-19 in 188 countries (1). People across the globe have experienced great life-changing events as a result of the disease's high morbidity and mortality (2). On January 30, 2020, the World Health Organization announced that COVID-19 was a public health emergency of international concern (2). The government took immediate action to restrict the spread of COVID-19 and encouraged people to take initiative to prevent a pandemic (3). Multimedia was utilized, such as television, social media, newspapers, and online websites, to inform people about the details of the disease, including potential symptoms, precautionary measures, and supportive services (4, 5). Individuals, concerned about contracting the disease and overcoming the uncertainty caused by conflicting information, began to search online and offline media for related information to keep themselves informed about COVID-19 (6–8). Hence, people were exposed to a vast amount of information.

The rapid spread of COVID-19 information resulted in an escalation of destructive effects (1). Further, accessing and processing a huge amount of information in a limited time can be burdensome and stressful, causing information overload (9). Meanwhile, some information sources lacked rigorous management of the accuracy and truthfulness of COVID-19-related information (10–12). This left individuals to filter misleading information, conspiracy theories, which exacerbated information overload (10).

Information overload was first defined by Toffer in the 1970s and has been investigated by many scholars (13). Information overload is defined as a situation in which the volume of information is beyond an individual's coping capacity (14). Prior research confirmed that information overload can cause individuals to become cognitively burdened and dysfunctional (15). When it becomes clear to an individual that he or she can no longer process a large amount of complex information, he or she will attempt to enhance coping abilities, which can be accompanied by stress, anxiety, depression, and feelings of being overwhelmed (2, 16). Furthermore, in the context of COVID-19 pandemic, the rapid dissemination of the false information, conspiracy theories amplified the negative emotions stem from COVID-19 information overload (17).

There are several empirical studies that revealed the relationship between COVID-19 information overload and anxiety (9, 18–20). Since the outbreak of COVID-19, characters have accessed a huge amount of COVID-19-related information, which is an energy-draining experience for those who do not possess deep prior medical knowledge to filter reliable and validate information from a large volume of information and make full sense of this information. While individuals are aware of the inability to make sense of perceived information, they might feel anxiety (9). Meanwhile, the wealth of information on websites about the diagnosis and symptoms of COVID-19 may mislead individuals to believe they are infected, even though some symptoms are common to multiple diseases, which inadvertently contributes to individual anxiety (9). For incidence, Saira et al. indicated the linkage between the source of COVID-19 information, COVID-19 information overload, and information anxiety, confirming that COVID-19 information overload is a strong predictor of anxiety (13).

Furthermore, during the New Coronary Pneumonia Pandemic, the social distance and isolation required by epidemic prevention policies can result in a wide range of negative emotions in individuals, such as depression, sadness, and loneliness (1). To buffer these uncomfortable feelings stemming from isolation and staying connected to the outside world, individuals tend to engage in social media or online resources much more, which exacerbates the extent of COVID-19 information overload (18). However, the amount of intricate information available in a short period of time is difficult for the individual to process, resulting in stress, which is a key factor of depression (10). Several empirical studies have also confirmed the relationship between COVID-19 information overload and depression. For example, a study conducted in Hong Kong indicated that higher COVID-19 information overload scores showed more severe depression symptoms (10). Moreover, Matthes tested the association between information overload and depression in a two-wave panel study and confirmed that there is a longitudinal relationship between information overload and depression (21). Numerous studies have examined the adverse outcomes of COVID-19 information overload, such as negative emotions, information fatigue, and information avoidance (2, 6, 18, 22). However, the adverse effects of COVID-19 information overload go far beyond these responses. The rapid spread of COVID-19 as well as the high mortality rate result in a negative psychological state, trigger an individual's stress, causing individuals to experience a variety of negative emotions, such as anxiety and depression (23, 24). When symptoms of acute stress disorder as negative emotions do not receive timely intervention, it may further occur as posttraumatic stress disorder(PTSD) (25).

In the context of COVID-19 pandemic, large amount of people was infected, caused not only the rise of disease burden as well as economic loss, but also psychological issues, especially the onset of posttraumatic stress disorder (PTSD) (26). PTSD was defined as delayed and prolonged psychiatric disorders following catastrophic or threatening trauma events to the individual. The disorder can be characterized by flashback, persistent avoidance, heightened alertness, selective amnesia, traumatic memories and loss of confidence (27), which is a severe life disrupting mental disorder. PTSD can lead to social dysfunction in individuals, causing seriously impairs quality of life, increasing the burden of disease on families and society (28–30).

There is evidence that negative emotions such as anxiety and depression are associated with PTSD. Empirical studies have shown that a variety of negative emotions, such as anxiety, depression, guilt, anger and depression, are associated with PTSD (31, 32). The established literature has clearly indicated that a high level of COVID-19 information overload has negative psychological and physiological consequences (6, 10, 13). Furthermore, more negative emotions predict the symptoms of PTSD (33). Therefore, it is likely that high levels of COVID-19 information overload indirectly affect the severity of PTSD symptoms by affecting anxiety and depression levels. Investigating the relationship between COVID-19 information overload, negative emotions and PTSD symptoms can deepen the understanding of PTSD in the context of COVID-19 pandemic, providing theoretical basis for psychological intervention in each key node.

However, whether COVID-19 information overload contributes to the occurrence of PTSD symptoms is still unknown. There are limited studies that have investigated the relationship between the variables. Hence, this study aimed to clarify the relationship between COVID-19 information overload and negative emotions such as anxiety and depression and PTSD symptoms, ensuring that accurate preventative measures can be offered at every key node in this process of negative changes in psychological states.

## Methods

### Study Design

A cross-sectional study carried out by 1150 subjects

### Subjects

This study was conducted from July 2020 to March 2021. A total of 1,302 subjects volunteered to participate in the study and were included. The inclusion criteria were as follows: (1) older than 18; (2) no cognitive impairment, for incidence, individuals who with severe dementia, in clouded consciousness, into stupor, in coma, fail to communicate due to severe psychiatric disorders or vegetative state were excluded; and (3) no severe somatic diseases, for incidence, those who currently suffer from cancer, acute trauma, shock were not include in this study. The exclusion criteria were as follows: (1) Not willing to participate in this study. The initial sample size (1067) was calculated by the formula N = Z^2^× (P × (1-P))/E^2^ (Z = 1.96, E = 3%, and *P* = 0.5). Due to the possibility of sample dropout, 1302 subjects were invited to finish the survey. Finally, 1150(88.32%) individuals finished the study. Those who withdrew from the study in the middle stage, for incidence, unable to continue this study due to personal matters, unable to understand the content of the questionnaires, and the answer sheets not available due to poor quality were removed from this study (235, 11.68%).

### Data Collection

**Figure 1:**
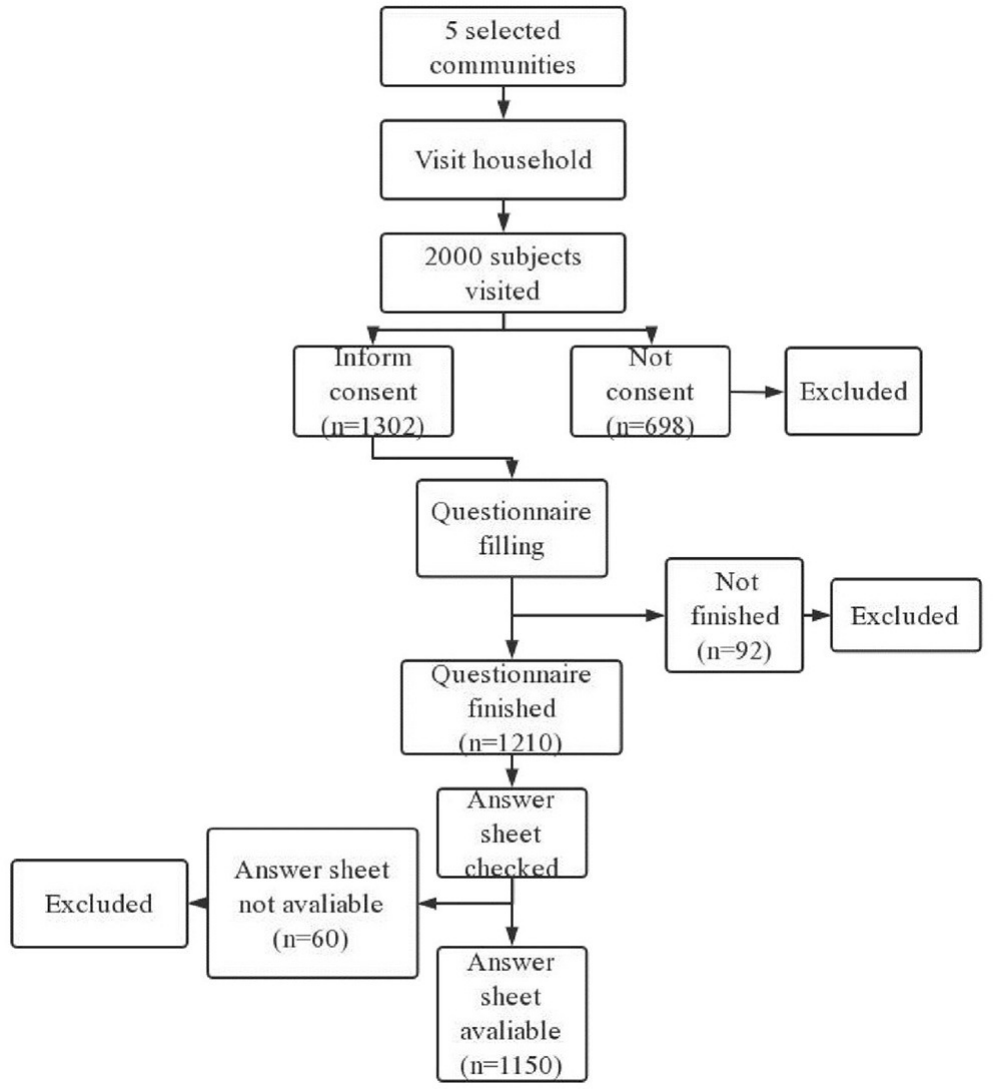
Sampling flow chart.

Before data collection, five communities were selected(Guojia Bridge Community, Nanhong Village Community, Shuangnan Community, Tangmen Street Community, Parachute Tower Community). The five community were all located in Wuhou District, with a total resident population of over 5,000 citizens. Leaders of three of the selected communities(Guojia Bridge Community, Tangmen Street Community, Parachute Tower Community) agreed to participate in this study. Data were collected by three well trained researchers. A WeChat message including the research proposal, precautions, and questionnaires was sent to each selected participant. Then, an online questionnaire was conducted to collect data. The most commonly utilized software, WJX (www.wjx.com), was used. Participants responded to questions about COVID-19 information overload, anxiety, depression, and symptoms of PTSD. In order to make sure the accuracy of each answer sheet as well as reduce bias, strict quality control measures were adopted. For example, each questionnaire cannot be submitted until all the questions were answered. One person could only fill in the questionnaire once after all the topics were completed. Each questionnaire was screened by automatic screening rules and manually checked by the researchers after submission. Any answer that did not meet the requirements, such as only one option was selected or the questionnaire was finished within 60 s, was marked as invalid and then removed. The details of the process of sampling shown in (Figure 1).

### Measurements

Mature measurements were adopted to ensure the accuracy of the study and the severity of information overload regarding text about the COVID-19 pandemic (34). The 7-item anxiety scale (GAD-7), the 9-item Patient Health Questionnaire depression module (PHQ-9); the psychometric properties of the PTSD Checklist (PCL) were utilized to measure individuals' COVID-19 information overload, anxiety, depression, and symptoms of PTSD.

#### The Information Overload Severity Scale on Text About the COVID-19 Pandemic

The scale was developed by Yang et al. in 2021 and consists of seven items measured on a five-point Likert scale, with higher scores representing more severe information overload (34). The scale has good reliability and validity, and previous study indicated that the total Cronbach's α coefficient of the scale was 0.863 (34). The total Cronbach's α coefficient of the scale in this study was 0.867.

#### The 7-Item Anxiety Scale (GAD-7)

The GAD-7 scale is a simple and effective assessment tool for the identification of generalized anxiety disorder. It is widely used overseas with a sensitivity of 86.8% and a specificity of 93.4%. The scale includes both somatic and cognitive/emotional scores, which are sensitive and simple to use (35). GAD-7 scores of 5-9, 10-14, and 15-21 represent mild, moderate, and severe anxiety, respectively (35). The total Cronbach's α coefficient of the scale in this study was 0.922

#### The 9-Item Patient Health Questionnaire Depression Module (PHQ-9)

The PHQ-9 scale was developed by Columbia University in the mid-1990s and is a self-assessment scale specifically designed to screen for mental disorders in primary health care settings (36). The PHQ-9 scale is more streamlined than other scales, and scores of 5–9, 10–14, 15–19, and 20–27 represent mild, moderate, moderate-severe, and major depression, respectively (37). The total Cronbach's α coefficient of the scale in this study was 0.919.

#### The PTSD Checklist-Civilian Version(PCL-C)

The PCL-C is a 17-item PTSD symptom questionnaire developed in November 1994 by the Behavioral Sciences Division of the American Center for PTSD Research and based on the DSM-IV (38). The PCL-C scale is designed to evaluate the experiences of ordinary people after experiencing trauma events in ordinary life (as opposed to war). It asks subjects to rate themselves according to how much they have been disturbed by problems and complaints in the past month on a five-point Likert scale, with higher scores representing more severe PTSD symptoms. The total score of each item was summed to determine the presence and severity of PTSD. In the United States, the PCL-C scale is often used as an evaluation scale for the diagnosis of PTSD symptoms and the evaluation of the effectiveness of interventions for the treatment of PTSD (39). The total Cronbach's α coefficient of the scale in this study was 0.947. PTSD was considered to be present when the total score exceeded 50 (40, 41).

### Ethical Consideration

Prior to conducting this study, an application was submitted to the Ethics Committee of West China Hospital Sichuan University. All the details about the research, as the research methods, measurements that intended to be utilized in this study, inclusion as well as exclusion criteria, research procedure were clarified in the application. After carefully review by the Ethics Committee of West China Hospital Sichuan University, the study obtained approval to conduct (No. K202006). All the participants were informed of all the details of this study and the signed informed consent form were obtained.

### Hypothesis and Research Model

To clarify the relationship between COVID-19 information overload and negative emotions and PTSD symptoms and investigate whether anxiety and depression have a mediation role between COVID-19 and PTSD symptoms, the following hypothesis were presented:

H1: COVID-19 information overload is positively associated with perceived anxiety and depression.

H2a: Anxiety is positively associated with an individual's PTSD symptoms.

H2b: Depression is positively associated with an individual's PTSD symptoms.

H3a: COVID-19 information overload indirectly affects the severity of PTSD by affecting anxiety.

H3b: COVID-19 information overload indirectly affects the severity of PTSD by affecting depression.

H3c: COVID-19 information overload is positively associated with individuals' PTSD symptoms.

**Figure 2:**
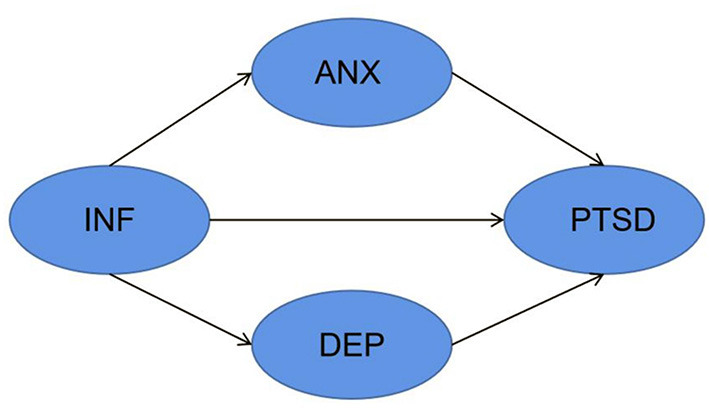
Research model. INF, COVID-2019 information overload; ANX, anxiety; DEP, depression; PTSD, post-traumatic stress disorder.

A research model was constructed to test the hypothesis above (Figure 2).

### Data Analysis

Since the purpose of this study is to test the proposed hypothesis, the Structural Equation Model was utilized. Structural equation model (SEM) is a statistical methods which is substantially utilized in psychology and social science. Compare to the other analyze strategies, SEM allows for confirmatory analyze, offering a comprehensive approach to evaluate and revise the theoretical model. Furthermore, SEM can also facilitate researchers to conduct simultaneous multiple mediating effects analysis, multiple mediation variables can be analyze simultaneously (42). Compare to the analysis of simple mediating effects, the total mediating effect can be obtained by utilizing SEM to establish multiple mediating effects. In addition, the mediating effect of each specific variable can be investigated with a premise that the other mediating variables are controlled. Comparative mediating effects can also be obtained by utilizing SEM, making it possible for the researchers to determine which mediating variable has a stronger effect (43). Therefore, the Structural Equation Model was utilized in this study. Before SEM conducted, the correlation between demographic factors and PTSD symptoms were tested, with a significance level of *p* < 0.05. Only demographic variables that were associated with PTSD symptoms were considered to be controlled.

IBM AMOS software 23.0 was adopted for data analysis. Before the path analysis, we conducted an initial screening to check the normality of the data. The bootstrapping procedure was utilized since the data in this study did not conform to a normal distribution. The two-step approach proposed by Anderson and Gerbing was adopted to test the research model as well as all the hypotheses (42). In the first step, model fit indices were utilized to check the degree of model fit. Further, the quality of the model was reflected by reliability and validity. The discriminant validity, convergent validity, construct reliability were tested (42). Discrimination validity was analyzed by calculating the correlation coefficient, which indicated the difference between two factors. Convergent validity was analyzed by testing the average variance explained (AVE). AVE is used to calculate the average variance explained by each measurements of the latent. Construct reliability was analyzed by composite reliability (CR). This indicator is a measurement of the degree of consistency of potential variables (42). In the second step, the path coefficient (β) and coefficient of determination (R^2^) were tested to examine the relationship between variables. In addition, the mediating roles of anxiety and depression were also analyzed by using a bootstrapping procedure with a sample size of 5,000 and a significance level of 0.05. The model was adjusted by modification index.

## Results

### Demographic Characteristics

There are 1150 subjects participated in this survey,including 410 male (35.7%), 740 female(64.3%), mean age 37.70 ± 13.91. 95(8.3%) participants were junior high school education experience and below, 151(13.1%) participants are senior high school educated. Most of the participants (796, 69.2%) has a college degree. 62.8%[722] of the participants are married. 42(3.7%) subject's neighborhoods had been infected by COVID-19. 12 (1.04%) of the participates were considered to be suspected or confirmed cases of COVID-19. Neighborhoods infected status was indicated to be related with individual's symptoms of PTSD (Table 1).


(1) $$\begin{array}{r} N=Z^{2}\left\{P\times \left(1-P\right)\right\}/E^{2} \end{array}$$

**Table 1:** Demographica characteristics among participants.

**Characteristics**	**N(%)**	**Z(H)**
**Gender**		
Male	410(35.7)	−0.341
Female	740(64.3)	
**Age**		
< = 27.00	306(26.6)	3.249
28.00–34.00	270(23.5)	
35.00–49.00	292(25.4)	
50.00+	282(24.5)	
**Educational background**		
Junior high school and below	95(8.3)	1.068
Senior high school	151(13.1)	
College degree	796(69.2)	
Bachelor degree and above	108(9.4)	
**Marital status**		
Unmarried	340(29.6)	3.790
Married	722(62.8)	
Divorced and other	88(7.6)	
**Neighborhoods infected status**		
Yes	42(3.7)	−2.884^***^
No	1108(96.3)	
**Being considered as suspected or confirmed cases of COVID-19**		
Yes	12(1.0)	−0.903
No	1138(99.0)	

*** *Indicated that p <0.005*.

### Individuals' COVID-19 Information Overload, Anxiety, Depression, and PTSD

According to the study, 36.0% had moderate anxiety, and 13.3% had severe anxiety. A total of 438(38.1%) of subjects had a moderate level of depression, 213 of the participates (18.5%) suffered from moderate-severe depression, 109(9.5%) suffered from major depression. Twenty-six subjects (2.3%) were considered to positive for PTSD screening, which indicated that the 26 participates may suffer from PTSD (Table 2).

**Table 2:** Individual's COVID-2019 information overload, anxiety, depression, PTSD status.

**Variables**	**Mean ±SD**	**Severity**	**(N,%)**
COVID-2019 information overload	8.82 ± 5.23	
Anxiety	10.61 ± 5.04	Mild	583 (50.7)
		Moderate	414 (36.0)
		Severe and above	153 (12.3)
Depression	12.89 ± 4.84	Mild	390 (33.9)
		Moderate	438 (38.1)
		Moderated-severe	213 (18.5)
		Major	109 (9.5)
PTSD	24.4 ± 8.87	Non PTSD	1124 (97.7)
		Positive for PTSD	26 (2.3)

*PTSD, Post-Traumatic Stress Disorder*.

### Measurement Model

The model fit was evaluated using different goodness-of-model fit indices. A measurement model was used by confirmatory factor analysis (CFA). To confirm good model fit, different thresholds were proposed by scholars: chi-square/degree of freedom (X2/df) <3, Comparative Fit Index (CFI) ≥ 0.95, Tucker–Lewis Index (TLI) ≥ 0.95, and Root Mean Square Error of Approximation (RMSEA) ≤ 0.05 (44). In our study, the measurement model indicated good fit (X2/df = 1.185, P = 0.210, CFI = 0.999, TLI = 0.999, RMSEA = 0.013, GFI = 0.995).

### Validity and Reliability

#### Discriminant Validity

Discriminant validity refers to the extent to which study constructs are significantly different from each other. Different threshold values were proposed by researchers to determine whether sufficient discriminant validity exists. The Fornell-Larker criterion and the inter-measurement correlation were tested (45). According to the Fornell-Larker criterion, the value of the inter-measurement correlation is supposed to be smaller than the square root of the average variance explained (AVE) for each research measurement. According to Brown et al., the correlation between each variable should not be >0.8. This is essential to distinguish significantly between any two given measurements because possible redundancy as well as possible covariance between the two given measurements might exist if the correlation value exceeds this threshold (46). The measures in this study fulfilled all these criteria, indicating sufficient discriminant validity of the study (Table 3).

**Table 3:** Mean, Standard deviation, convergent and discriminant validity.

**Variables**	**MEAN**	**SD**	**CR**	**AVE**	**INF**	**ANX**	**DEP**	**PTSD**
INF	8.82	5.23	0.850	0.653	**0.808**			
ANX	10.61	4.04	0.917	0.787	0.61	**0.887**		
DEP	12.89	4.84	0.914	0.781	0.487	0.704	**0.884**	
PTSD	24.4	8.77	0.910	0.772	0.566	0.696	0.747	**0.877**

*INF, COVID-2019 information overload; ANX, anxiety; DEP, depression; PTSD, post-traumatic stress disorder*.

*CR, Composite Reliability*.

*AVE, Average Variance Explained. Bold values means factor loading of the variables*.

#### Convergent Validity

The degree to which a measure reflects the same essential concept indicates convergent validity. Different standards to verify the existence of sufficient convergent validity were proposed by researchers, as the factor loadings should be higher than 0.70 (47). The AVE values are supposed to be higher than 0.50, indicating that a given metric possesses at least half of the variance to be explained (47). The measures in this study fulfilled all these criteria, indicating sufficient convergent validity of the study (Table 4).

**Table 4:** Cronbach's α statistics, loadings of the study.

**Study measures**	**Measurement items**	**Loading**	**Cronbach' αstatistics**
COVID-2019 Information overload (INF)	INF1	0.857	0.867
	1. In the last month, have you received so much information about the pandemic that it has “overwhelmed” you?		
	6. In the last month, have you feel stressed because you received a lot of information related to the pandemic from different sources in a short period of time?		
	7. In the last month, have you feel that the COVID-2019 information you received at once was more than you could handle?		
	INF2	0.765	
	2. In the last month, did you forget to reply to a very important message?		
	5. In the last month, do you have to spend more time maintaining your communication devices in order to receive information?		
	INF3	0.801	
	3. In the last month, have you felt that you have to constantly refresh of search COVID-2019 related information?		
	4. In the last month, did you receive more COVID-2019 information than you could handle?		
Anxiety (ANX)	ANX1	0.923	0.922
	4.Feeling it is hard to relax yourself		
	5.Unable to meditation due to restlessness		
	7.Feeling worried because something terrible seems to be going to happen		
	ANX2	0.863	
	2.Unable to stop worrying		
	3.Worrying too much about various things		
	ANX3	0.876	
	1.Feeling nervous or anxious		
	6.Feeling easily annoyed or impatient		
Depression (DEP)	DEP1	0.836	0.919
	1.Feeling unmotivated or uninterested in what you are doing in the last 2 weeks		
	2.Feeling down, frustrated or hopeless in the last 2 weeks		
	3.Difficulty in falling asleep, sleeping restlessly, or sleeping excessively		
	DEP2	0.922	
	4.Feeling fatigue in the last 2 weeks		
	5.You have lost your appetite or eaten too much in the last 2 weeks		
	6.You feel bad about yourself, or feel like a failure in the last 2 weeks		
	DEP3	0.878	
	7.You have trouble focusing on things, such as reading the newspaper or watching TV in the last 2 weeks		
	8.People can notice that you are talking or moving significantly slower than before, or conversely, you seem more irritable than usual in the last 2 weeks		
	9.You sometimes think it's better to die or you have the thoughts to hurt yourself in the last 2 weeks		
PTSD	PTSD	0.986	0.947
	• Did the pandemic has brought back uncomfortable memories, thoughts or images to you repeatedly?		
	2. Did the pandemic caused you recurring nightmares?		
	3. Did you feel as if the pandemic has broken out again or gotten worse?		
	4. Did you feel restless if something remind you about the pandemic?		
	5. Did you feel somatic discomfort if something remind you about the pandemic?		
	PTSD	0.859	
	6. Did you try to avoid thinking of or talking past experience about the pandemic?		
	7. Did you try to avoid participating in the events that remind you about the pandemic?		
	8. Did you have trouble remembering important information about the pandemic?		
	9. Did you loss interest in things you used to enjoy?		
	10. Did you feel alienate from others?		
	11. Did you feel emotional numbness?		
	12. Did you feel uncertain about you future?		
	PTSD3	0.939	
	13. Did you have trouble to fall asleep, or easily to wake up?		
	14. Did you feel that you are more easily to lose your temper than before?		
	15. Did you have trouble to concentrate?		
	16. Did you are very alert?		
	17. Did you feel easily frightened?		

*INF, COVID-2019 information overload*.

*ANX, anxiety*.

*DEP, depression*.

*PTSD, post-traumatic stress disorder*.

#### Construct Reliability

Composite reliability (CR) was tested to investigate the construct reliability of these study measures. Adequate construct reliability was established when the CR exceeded 0.70, according to Clark and Watson (48). The measures in this study fulfilled all these criteria, indicating composite reliability (Table 3).

### Structural Model

**Figure 3:**
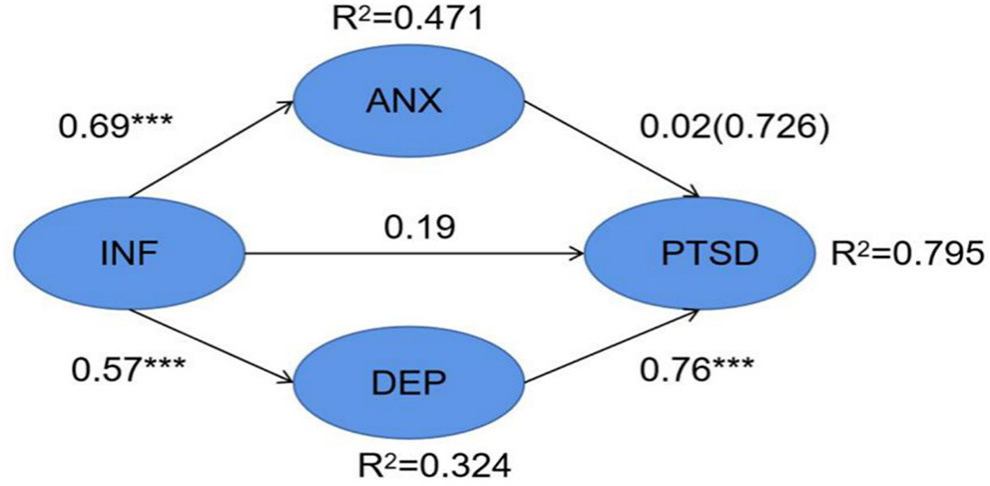
Structural Model results showing path coefficients and coefficients of COVID-2019 information overload, anxiety, depression, PTSD. ***p < 0.001.

The structural model was evaluated using structural equation modeling (SEM). Through this approach, we investigated the relationship between information overload as a condition prior to anxiety, depression, and PTSD and the effect of anxiety and depression on PTSD. Different research hypotheses were developed according to the size and significance of the structural paths. In addition, the squared multiple correlation (R^2^) values were tested to ascertain the proportion of variance explained in the dependent variable. We identified the potential correlations of the study measures on the basis of significance levels and squared multiple correlation values. The path coefficients and significance levels are given in Figure 1 (Figure 3). The R^2^ values of anxiety, depression, and PTSD were 0.471, 0.324, and 0.795, respectively, indicating that 47.1% of the variance in anxiety, 32.4% of depression, and 79.5% of PTSD can be explained. COVID-19 information overload was positively associated with anxiety (β=0.687, *p* < 0.001), depression (β=0.569, *p* < 0.001), and symptoms of PTSD (β=0.190, *p* < 0.001), and depression was positively associated with symptoms of PTSD (β=0.757, *p* < 0.001). Therefore, H1, H2b, H3c accepted, H2a rejected.

### The Mediating Effect of Anxiety and Depression

To confirm whether COVID-19 information overload indirectly influences individuals' PTSD symptoms by affecting anxiety and depression, the bootstrap method was adopted. We defined special syntax to perform the analysis of mediating effects; ind1 indicated the indirect effects of COVID-19 information overload which mediated by anxiety, and ind2 indicated the indirect effects of COVID-19 information overload which mediated by depression. The direct effect of COVID-19 information overload and total effects or the sum of ind1, ind2, direct effect were also calculated (Table 5). According to the mediating analyze, H3b accepted, H3a rejected.

**Table 5:** Mediating effect, direct effect and total effect t.

**Mediating effect**	**?**	**95%CI**	**P**	**B (standard)**	**95%CI (standard)**
Ind1-anxiety	0.020	−0.143–0.168	0.810	0.012	
Ind2-depression	0.738	0.597–0.912	<0.001^***^	0.430	
Direct effect	0.325	0.211–0.464	<0.001^***^	0.190	0.122–0.268
Total effect	1.083	0.950–1.236	<0.001^***^	0.632	0.576–0.685

*Ind1, the indirect effects of COVID-2019 information overload which mediated by anxiety*.

*Ind2, the indirect effects of COVID-2019 information overload which mediated by depression*.

## Disscussion

This study provides novel insights associated with COVID-19 information overload and its impact in the pandemic context. The antecedents, measurements, incidence rates, causes, and effects of COVID-19 information overload on mental health are widely studied (2, 6, 9, 10, 22); however, in addition to negative emotions and psychological illness, chain effects need to be considered. Therefore, according to previous studies that indicated a relationship between COVID-19 information overload and anxiety, depression, and PTSD symptoms (9, 10, 49, 50), we investigated the relationships among these variables. We found that COVID-19 information overload has a small direct effect on the development of PTSD symptoms; moreover, COVID-19 information overload can affect the development of PTSD symptoms indirectly by affecting an individual's self-reported depression, which offers a new perspective on interventions for PTSD. The results of the study also confirmed that COVID-19 information overload was positively associated with anxiety and depression, which is in accordance with previous studies (2, 10). As a small psychological discomfort can subsequently turn into a serious mental illness, our findings have clinical implications (21).

The results of the study indicated that depression mediated the process of COVID-19 information overload leading to PTSD symptoms. According to Matthes's findings, COVID-19 information overload can be a trigger of depression (21). A large amount of information on a wide range of topics appears on websites and forums, and comments on this information, which exceed individuals' information processing ability, can result in frustration and lead to depression (9). Moreover, depressed individuals are more inclined to spend much more time on social media and online information resources, which forms a vicious cycle (51). When depression accumulates it may lead to PTSD. An investigation conducted by Breslau *et al*. showed that there may be multiple relationships between PTSD and depression: patients with PTSD and those with depression have similar personality traits. PTSD and depression may also be causally related to each other (52). According to Yaacoub's study, the relationship between depression and PTSD can be explained by feelings of insecurity, which lead to a more distorted memory of the event and to more intense emotions and hurt. This aggravation of an already distressing trauma increases the chances of developing PTSD (49). Therefore, COVID-19 information overload might affect individuals' PTSD symptoms through the psychological pathways mentioned above.

In this study, we also found a small direct association between COVID-19 information overload and PTSD symptoms. According to the symptoms of PTSD, when PTSD patients are exposed to information related to a traumatic event or an environment that resembles the traumatic event, they experience intense feelings of discomfort, such as fear, panic, and depression (53). Therefore, the widespread circulation of COVID-19-related information might lead to increased exposure to events or environments related to traumatic memories. In addition, we deduce that the infodemic of fake news, conspiracy theories, and polarizing information might amplify feelings of insecurity, which is an essential sensation that causes PTSD. Hence, COVID-19 information overload may directly affect PTSD levels in these ways.

In our study, the relationship between COVID-19 information overload and anxiety and depression was also validated. In accordance with prior studies, COVID-19 information overload is positively associated with anxiety and depression (10). Therefore, COVID-19 information overload is an important contributor to the negative impact on people's psychological states in the short or long term (10). The rapid rise in new cases of COVID-19 around the world and the subsequent changes in everyday living are causing panic and stress (23, 54). Information overload and rumor spreading during the pandemic added additional psychological burden to the public, affected the risk perception of individuals, decreased confidence in fighting the disease, and became a catalyst for disrupting individuals' well-being (55). If timely action is not taken to provide the public with psychological guidance, the accumulation and fermentation of negative emotions will cause deeper damage from the initial temporary negative emotions to the development of serious PTSD symptoms (55). In this process of negative changes in psychological states, each node is the key to psychological prevention, and effective measures should be taken to mediate the chain-like deterioration of individuals' well-being.

It is interesting that anxiety is not associated with PTSD symptoms. According to prior studies that explored the relationship between depression and anxiety, anxiety could turn into depression (56). At the end of 2019, during a long period of isolation, anxiety stemming from COVID-19 information overload might turn into depression, and subsequently PTSD. However, in our findings, anxiety was not an influencing factor of PTSD.

This study has significant theoretical and practical implications. There are significant theoretical practical implications in this study. First, our findings are in accordance with the increasing number of studies and the recommendations of the WHO to reduce exposure to COVID-19-related information (10). Second, the linkage of PTSD and COVID-19 information overload has not yet been well examined. Therefore, the research is possibly the first empirical study to our knowledge that has examined them, which promotes the theoretical development of COVID-19 information overload. Third, the outcomes or consequences of COVID-19 information have been well investigated in relation to anxiety, depression, stress, information avoidance, etc. (6, 13); however, it is still unclear what are the further psychological outcomes. Minor psychological discomfort could develop severe mental disorders such as PTSD (57). This could be an opportunity for the development of future therapeutic interventions that could be delivered or partially delivered by mitigating COVID-19 information overload and, in return, alleviating the amplified negative outcomes among this population.

Our study has several practical implications. This study verified the relationship of PTSD, anxiety, depression and COVID-19 information overload. Hence, effective measures need to be adopted to reduce the extent of COVID-19 information overload if we want to reduce the incidence of anxiety, depression, PTSD or other negative outcomes (2). First, the chain linkage of COVID-19 information overload, depression, and PTSD suggested the significance of timely intervention at each point of mental state transformation. The mediating role of depression and PTSD could be a specific guideline for psychological intervention and prevention under the context of COVID-19 infodemic. Second, our findings warrant policy makers that there is a need for public training to help them learn the criteria for determining the credibility of information on various platforms (2). Medium reliability (58), origin reliability (59), and message reliability (60), which are three proven influencing elements, are supposed to be involved in the training. Third, the findings reminded technology companies or other related stakeholders to manage the quality of COVID-19-related information, providing accurate and true information (10). Fourth, practical guidelines for individuals to cope with major crises are needed; otherwise, they might produce cognitive dysfunction, psychological disorders, and affective pressures (2).

This study has several limitations. First, this is a cross-sectional study that is unable to observe the transformation of depression into PTSD. Hence, further longitudinal studies and randomized controlled trials are needed to confirm the findings. Second, the measurements adopted in our study are brief screenings for depression, anxiety, and PTSD. Further clinical interviews and diagnoses are indispensable, as there is potential bias of self-rating scales. Third, the scales that measure the extent of COVID-19 information overload were developed and adopted in China, and the results might not be fully applicable to Western countries. Analysis of reliability and validity among different cultural contexts is warranted. Furthermore, the results of this study should also be tested in other countries.

## Conclusion

The purpose of this study was to understand the short-term and long-term psychological outcomes of COVID-19 information overload during the pandemic in China. This study empirically explored the relationship between COVID-19 information overload, anxiety, depression, and PTSD. The findings of our study revealed a mediating role of depression in the process of COVID-19 information overload leading to PTSD. The positive association of COVID-19 information overload with anxiety and depression was also confirmed, suggesting the need for psychological interventions at specific times. Practical public training, such as crisis coping and information filtering, is essential. Regulation of technology companies is also essential.

## Data Availability Statement

The original contributions presented in the study are included in the article/Supplementary Material, further inquiries can be directed to the corresponding authors.

## Ethics Statement

The studies involving human participants were reviewed and approved by the Ethics Committee of West China Hospital Sichuan University. The patients/participants provided their written informed consent to participate in this study. Written informed consent was obtained from the individual(s) for the publication of any potentially identifiable images or data included in this article.

## Author Contributions

JW and XH involved in design of the study, acquisition of data, development of the statistical framework and reviewed the manuscript. MW and XL involved in the study design of and development analysis framework. JX and YW developed the statistical framework for data analysis, conducted the statistical analysis, interpreted the data and drafted the manuscript. All authors contributed to the article and approved the submitted version.

## Funding

This research was supported by the Science & Technology Department of Sichuan province fund project (Program No: 2021YFS0151), the Science & Technology Department of Sichuan province fund project (Program No: 2022JDKP0002), West China School of Nursing, Sichuan university (Program No: HXHL19012).

## Conflict of Interest

The authors declare that the research was conducted in the absence of any commercial or financial relationships that could be construed as a potential conflict of interest.

## Publisher's Note

All claims expressed in this article are solely those of the authors and do not necessarily represent those of their affiliated organizations, or those of the publisher, the editors and the reviewers. Any product that may be evaluated in this article, or claim that may be made by its manufacturer, is not guaranteed or endorsed by the publisher.
